# *Adlercreutzia*-modulated polyunsaturated fatty acid metabolism underlies nicotine’s anti-obesity effects

**DOI:** 10.3389/fmicb.2025.1682370

**Published:** 2025-12-18

**Authors:** Yifan Duan, Xiao Li, Ying Chai, Huan Chen, Hongwei Hou

**Affiliations:** 1 Qingdao University, Qingdao, China; 2 Beijing Life Science Academy, Beijing, China; 3 China National Tobacco Quality Supervision and Test Center, Zhengzhou, China

**Keywords:** obesity, gut microbiota, nicotine, metabolism, PUFA

## Abstract

**Background:**

The regulatory effects of nicotine on energy balance through central and peripheral mechanisms have been reported. However, its impact on obesity and gut microbiota at safe doses remains unclear.

**Results:**

In this study, it was found that chronic oral nicotine administration daily at relative low dose (0.5 mg/kg) significantly alleviated high-fat diet (HFD)-induced obesity phenotypes in mice, including body weight gain, fat deposits, hepatic steatosis, inflammation and metabolic dysfunction. Gut microbiota depletion and fecal microbiota transplantation (FMT) confirmed that these beneficial effects were microbiota-dependent. Metagenomic sequencing confirmed that nicotine administration reshaped gut microbiota composition, and specifically enriched the commensal genus *Adlercreutzia*, whose increased abundance correlated with improved biochemical indicators related to obesity. Furthermore, transplantation of *Adlercreutzia* reproduced anti-obesogenic effects, suggesting it was a key factor for nicotine reducing HFD-induced obesity. Untargeted metabolomics analysis combined association analysis further demonstrated that nicotine modulated host metabolic profiles via gut microbiota-metabolite axis, particularly enhancing *Adlercreutzia*-linked lipid metabolites involved in polyunsaturated fatty acid (PUFA) metabolism.

**Conclusion:**

Collectively, our study elucidates the critical involvement of gut microbiota in nicotine-induced obesity amelioration, uncovers a novel *Adlercreutzia*-PUFA metabolic axis mediating nicotine’s anti-obesity effects, and highlight *Adlercreutzia* potentiation as a promising microbiota-directed invention strategy for obesity and metabolic syndrome.

## 1 Introduction

Obesity, currently defined as a body mass index (BMI) greater than or equal to 30, has become a globally prevalent chronic disease characterized by excess adiposity with structural and functional consequences, leading to increased risk of various comorbidities including type 2 diabetes mellitus (T2DM), cardiovascular disease, osteoarthritis, sleep disorders, and multiple cancers (Elmaleh-Sachs et al., 2023; World Health Organization, 2023). So far, the main therapeutic strategies for weight reduction and obesity control are lifestyle interventions, anti-obesity medications, and endoscopic and bariatric surgeries or combination therapies (Elmaleh-Sachs et al., 2023; Kollins et al., 2009). However, these means have the disadvantages of weight regain, side effects and invasiveness, and there is an urgent need to find new strategies for the prevention and treatment of obesity and its associated comorbidities. Growing evidence indicated that obesity is associated with dysregulated metabolic and immune system (Gilbert et al., 2018). Thus, ameliorating obesity by modulating underlying immunometabolism may be an effective strategy.

The intestinal microbiota is now considered to be a new complex organ, consisting of 500–1,000 bacterial species with the amount up to 10^14^, and the number of genes in the intestinal microbiota is approximately 100 times of that in humans (Gilbert et al., 2018). The homeostasis of intestinal microbiota plays an important role in promoting human health, and the dysregulation of intestinal microbiota composition and function contributes to the development of various metabolic diseases, including obesity, T2DM, and non-alcoholic fatty liver disease (NAFLD) (Fan and Pedersen, 2021). The development of obesity is closely associated with gut microbiota dysbiosis. Specific gut microbial species, such as *Firmicutes* and *Bacteroidetes*, and their ratios, have been linked to the development of obesity in population (Ley et al., 2006). In particular, high-fat diets significantly alter the composition and function of the intestinal microbiota, typically leading to reduced microbial diversity, an increased *Firmicutes*-to-*Bacteroidetes* ratio, a decline in beneficial metabolite-producing bacteria such as short-chain fatty acids (SCFAs) (Turnbaugh et al., 2006). This diet-induced imbalance disrupts gut barrier integrity, triggers low-grade systemic inflammation, and disturbs host energy homeostasis, thereby exacerbating obesity and related metabolic disorders. Notably, gut microbial metabolites, especially SCFAs derived from dietary fiber fermentation, serve as key links between the microbiome, host metabolism, and immunity (Mann et al., 2024). SCFAs could mitigate obesity by regulating energy balance, protecting intestinal barrier integrity, and reducing intestinal inflammation (Patloka et al., 2024). Supporting the therapeutic potential of targeting the gut ecosystem, a clinical trial indicated that low-fermentable fiber supplementation following oral fecal microbiota transplantation (FMT) improved insulin sensitivity in patients with severe obesity and metabolic syndrome (Mocanu et al., 2021). These findings collectively highlight that modulating gut microecology represents a promising strategy for preventing and treating obesity.

Emerging evidence suggested several natural alkaloids could reduce obesity by regulating the composition and functions of individual intestinal microbiota. Animal studies have shown that betaine improves high-fat diet (HFD)-induced glucose and lipid metabolism by regulating gut microbiota dysbiosis and increasing anti-obesity strains as well as SCFAs production. Ramulus mori (Sangzhi) alkaloids (SZ-A), which has been approved for the treatment of T2DM in China, was also found to alleviate obesity-driven lipid metabolic disorders and inflammation through regulating gut microbiota disorder and its metabolism profiles (Liu et al., 2023). These findings highlight the potential of natural alkaloids as prebiotics to ameliorate obesity.

Nicotine is the most abundant alkaloid in tobacco, accounting for about 95% of the total alkaloid content of tobacco, and is also the main psychoactive substance in smoke (Sansone et al., 2023). Numerous epidemiological studies have shown a strong association between smoking, smoking cessation, and weight fluctuation, with current smoking negatively correlated with body weight and smoking cessation induced weight gain (Dare et al., 2017; Kaufman et al., 2012; Mackay et al., 2013; Sneve and Jorde, 2008). Further experimental evidence demonstrated that smoking and cessation induce gut microbial dysbiosis, and identified gut microbial metabolites such as dimethylglycine and N-acetylglycine as being associated with smoking-cessation-induced weight gain (Fluhr et al., 2021). The role of nicotine in the regulation of energy balance has been studied, involving both central and peripheral mechanisms. Nicotine reduces energy intake by inhibiting hypothalamic orexigenic AgRP/NPY neurons and activating anorexigenic POMC neurons (Jang et al., 2003; Mineur et al., 2011), and increases energy expenditure by inhibiting hypothalamic AMPK (Martínez de Morentin et al., 2012), activating sympathetic nervous system (Mano-Otagiri et al., 2009) and increasing uncoupling protein 1 (UCP1) expression (Arai et al., 2001) to promote thermogenesis in brown adipose tissue (BAT). In addition, nicotine stimulates adipocyte lipolysis (Andersson and Arner, 2001) and regulates glycemia and insulin sensitivity (Vu et al., 2014). These evidence suggest that nicotine regulates energy metabolism through a variety of biological processes, but most of these studies were not conducted in the context of obesity, and the relationship of nicotine administration, energy metabolism and obesity is unclear.

The interaction of nicotine with gut microbes has also been revealed. Nicotine accumulates in the intestine during tobacco smoking, gut bacterium *Bacteroides xylanisolvens* can degrade intestinal nicotine, thereby improving nicotine-exacerbated NAFLD progression (Chen et al., 2022). It has been shown that nicotine-induced weight suppression is associated with specific changes in gut microbial composition and metabolites, including the increase of *Lactobacillus spp*., and KetoB, a nicotine-sensitive metabolite of polyunsaturated fatty acid (PUFA) (Ohue-Kitano et al., 2024). Notably, in studies related to nicotine’s effects on energy metabolism and obesity amelioration, nicotine is administrated in different ways and the dosage varies widely (Qin et al., 2025). It is known that the administration mode and dosage of nicotine influence its biological effects, including effects on gut microbial composition and metabolites level (Ohue-Kitano et al., 2024; Qin et al., 2025). Therefore, further investigations of the modulatory effects of nicotine on obesity, obesity-driven metabolic disorders, and gut microbial status at different doses including safe doses are required, for better understand the interplays within the trifecta of intestinal microbiota, obesity and host metabolism.

In the present study, in order to explore the direct action of nicotine on intestinal microbiota, the mice were administrated nicotine by oral gavage at relative low dose. The results showed that chronic low dose of nicotine administration significantly reduced obesity characteristics and related metabolic disorders in HFD-induced obese mice. Metagenomic and metabolomics analyses revealed nicotine-induced alterations in intestinal microbiota composition and metabolism profiles of obese mice. Mechanistically, the anti-obesity benefits of nicotine were associated with an increase in the intestinal commensal *Adlercreutzia spp*. along with PUFA metabolites. In conclusion, our study revealed the mechanisms by which nicotine modulates intestinal microbiota, identified a novel intestinal commensal microbial species that mediates the anti-obesity effects of nicotine, and offered the possibility of its use as a potential health supplement for the prevention of obesity and related metabolic disorders.

## 2 Material and methods

### 2.1 Animal treatment

#### 2.1.1 Experimental animals

Male C57BL/6 male mice aged 6 weeks used in our study were purchased from Beijing Vital River Laboratory Animal Technology Co., Ltd., and maintained under specific-pathogen-free (SPF) conditions. Mice were fed 60 kcal% high-fat chow (Research Diets, United States) to establish high-fat diet (HFD)-induced obesity model. In all animal experiments, mice were housed at temperature of 22 °C ± 2 °C, humidity of 45%–65% and a light/dark cycle of 12 h with food and water *ad libitum*. At the experimental endpoint, blood was collected from the orbital sinus after anesthesia via intraperitoneal (i.p.) injection of 1.25% tribromoethanol (0.2 mL/10 g body weight, M2920, Nanjing Aibei Biotechnology), followed by euthanasia through cervical dislocation, followed by euthanasia via cervical dislocation. The schematic diagram of all animal experimental procedures in this study is shown in Supplementary Figure 1. All experiments were performed in accordance with the ethical policies and procedures approved by the Animal Care Welfare Committee of Zhengzhou University (ZZU-LAC20241009[01]).

#### 2.1.2 Nicotine treatment

To evaluate the effects of nicotine treatment on HFD-induced obesity, mice pre-fed a HFD for 8 weeks were randomly divided into two groups (*n* = 6) and, respectively gavaged daily with nicotine dissolved in saline (0.5 mg/kg) or saline as a control for 12 weeks, while maintaining a HFD. The preparation and administration procedure of nicotine was specified as follows: Pure nicotine (provided by China National Tobacco Quality Supervision and Test Center) was dissolved in saline to prepare a stock solution of 100 mg/mL. Before administration, it was diluted to a working solution of 0.1 mg/mL and sterilized by filtration through a 0.22 μm filter. A fresh solution was prepared for each administration. Mice were given nicotine or saline at a dose of 100 μL/20 g.

During the treatment, mice were regularly monitored and recorded for body weight changes, food and water intake. At the end of administration, mice were fasted for 12 h in advance with free access to water, and whole blood samples were obtained through orbital blood sampling. After being placed at room temperature (RT) for 1 h, the samples were centrifuged at 3,000 g for 15 min to collect serum, which was used for the detection of lipid, diabetes biomarkers and inflammatory factors. Then, the mice were sacrificed, fat tissues and other organs were removed, weighed and subjected to histopathological testing. Meanwhile, fecal samples were collected in cryotubes, snap-frozen in liquid nitrogen and stored at −80 °C for subsequent metagenomic sequencing. Metabolic parameters and omics profiles were measured for each mouse.

#### 2.1.3 Antibiotics (ABX) treatment

To determine whether the anti-obesity effects of nicotine are linked to the gut microbiota, mice were treated with ABX to construct a pseudo-sterile mouse model. Briefly, HFD-fed mice were randomized into two groups (*n* = 6) and administered nicotine (0.5 mg/kg) or saline for 4 weeks. During this period, mice were provided with sterile drinking water supplemented with an antibiotic cocktail containing 1 g/L metronidazole, 1 g/L ampicillin, 0.5 g/L vancomycin, and 1 g/L neomycin (MCE, China). Blood, tissue and fetal samples were collected and processed following methods similar to those described in see section “2.1.2 Nicotine treatment.”

#### 2.1.4 Fecal microbiota transplantation (FMT)

To further ascertain the role of the gut microbiota in the anti-obesogenic effects of nicotine, FMT was performed using fecal material obtained from HFD-fed mice from experiments “2.1.2 Nicotine treatment” that had received saline or nicotine administration. For the last 2 weeks of treatment, fresh feces from each donor mouse were collected daily using a sterile fecal collector, and then mixed with 10% glycerol at a ratio of 1:1 (W/V), aliquoted into sterile cryotubes, and immediately stored at −80 °C. When used, the frozen samples were slowly thawed at RT and prepared in anaerobiosis. Fecal samples were collected and transported under anaerobic protection. Immediately after collection, feces were placed into anaerobic transport bags containing oxygen-absorbing agents (AnaeroPack, MITSUBISHI GAS CHEMICAL COMPANY, INC.). All procedures for preparing fecal suspensions were performed in an anaerobic chamber (Bactron EZ-2, Shellab, United States) filled with a mixed anaerobic gas atmosphere (10% H2, 10% CO2, and 80% N2). All solutions used for suspension and dilution (including saline and glycerol) were deoxygenated and pre-equilibrated in the anaerobic chamber before use. Freshly collected fecal samples were promptly transferred into the anaerobic chamber for weighing, suspension, and homogenization to ensure the viability and activity of oxygen-sensitive gut microbes. Feces from different mice in the same group were pooled together and mixed with sterile saline at a 1:10 (W/V) ratio, then vortexed to homogeneity. After centrifugation at 500 g for 5 min at 4 °C, the supernatant was taken for transplantation. HFD recipient mice pre-treated with antibiotics for 2 weeks were randomized into two groups (*n* = 6), and gavaged with the fecal microbiota transplants from each donor group at a dose of 100 μL/20 g, once a day for the first 3 days and then twice a week for a total of 8 weeks. Blood and tissue samples were collected and processed similarly as described in see section “2.1.2 Nicotine treatment.”

#### 2.1.5 *Adlercreutzia* treatment

*Adlercreutzia equolifaciens* (DSM 19450) was purchased from German Collection of Microorganisms and Cell Cultures. *Adlercreutzia* were cultured in chopped meat medium with carbohydrates (DSMZ medium 110, Germany), and placed in an anaerobic chamber (Shellab, United States) at 37 °C. When they reached the logarithmic growth phase, the concentration was determined by measuring OD_600_ using a UV spectrophotometer. After centrifugation at 6,000 g for 5 min, the bacteria pellet was resuspended in sterile PBS at 10^8^–10^9^ CFU/mL. HFD recipient mice pre-treated with antibiotics for 2 weeks were randomized into two groups (*n* = 6), and orally administrated active *Adlercreutzia* or inactived *Adlercreutzia* by pasteurization at a dose of 100 μL/20 g, twice a week for 8 weeks. Blood and tissue samples were collected and processed similarly as described in see section “2.1.2 Nicotine treatment.”

### 2.2 Detection of obesity indicators

#### 2.2.1 Bodyweight measurements

The body weight of each mouse was measured on days 1, 3, and 5 of each week using an electronic scale to monitor weight changes. The ratio of the increment of treatment bodyweight to initial bodyweight was calculated as body weight gain (%).

#### 2.2.2 Biochemical indices detection

Lipid marker levels in liver tissues and serum, including total cholesterol (TC), triglycerides (TG), low-density lipoprotein cholesterol (LDL-c) and high-density lipoprotein cholesterol (HDL-c) were measured using commercial colorimetric kits (Nanjing Jiancheng Bioengineering Institute, Nanjing, China). Concentrations of diabetes biomarkers and inflammatory factors in serum were determined using mouse diabetes multiplex immunoassay kit and cytokine multiplex immunoassay kit (Bio-Rad, United States).

#### 2.2.3 Oral glucose tolerance test (OGTT)

The OGTT was performed 2 or 3 days before the end of treatment. Mice were fasted for 12 h in advance with free access to water. Each mouse was gavaged with 50% glucose solution (Aladdin, Shanghai, China) at the dose of 2 g/kg, and blood glucose levels were measured by a glucometer (OneTouch, Shanghai, China) at 0, 15, 30, 60, 90, and 120 min post-gavage.

#### 2.2.4 Morphology analysis

Epididymal white adipose tissue (eWAT) and inguinal WAT (iWAT) were dissected from each mouse, cut into small pieces, fixed in 4% paraformaldehyde (PFA), dehydrated, cleared, paraffin-embedded, and sliced into 5 μm sections. The sections were subjected to hematoxylin and eosin (H&E) staining, images were captured using Pannoramic MIDI II Slide Scanning and Image Analysis System (3DHISTECH, Hungary), and the area of adipocytes was measured by Image J software.

#### 2.2.5 Oil Red O (ORO) staining

Liver tissues from each mouse were OCT-embeded, snap frozen at −80 °C, and then sliced into 8 μm sections. The sections were rinsed with distilled water and soaked in 60% isopropanol for 20–30 s. Next, the sections were stained using oil red (Servicebio, Wuhan, China) working solution for 10 min. After rinsed with 60% isopropanol to remove excess dye and immersed in distilled water to remove isopropanol, the sections were counterstained with Mayer’s hematoxylin solution (Servicebio, Wuhan, China) for 1–2 min to stain the nuclei. Images were observed and photographed using Pannoramic MIDI II Slide Scanning and Image Analysis System.

#### 2.2.6 Short-chain fatty acids (SCFAs) measurements

Urine supernatant was aliquoted for SCFAs analysis. After dilution, samples were sequentially added with ethanol, an internal standard, and NaOH. The mixture was vortexed and freeze-dried. The dried residue was reconstituted in ethanol and 1,4-butanediol. The contents of SCFAs, including Acetic acid, Propionic acid, Isobutyric acid, N-Butyric acid, Isovaleric acid, Valeric acid, Hexanoic acid, 2-Methylbutyric acid were quantified using an Agilent 8890-5977B GC-MS system.

#### 2.2.7 Quantitative real-time PCR (qPCR)

Total RNA was extracted from tissues using the Steady Pure Universal RNA Extraction Kit (Agbio, Hunan, China) and reverse transcribed into complementary DNA (cDNA) by Evo M-MLV RT Kit (Agbio, Hunan, China). qPCR analysis was performed with six biological replicates (*n* = 6), each measured in three technical replicates, using the SYBR Green Pro Taq HS Premix qPCR Kit (Agbio, Hunan, China) on the RT-PCR System (ROCHE, LightCycler96). Relative mRNA expressions of genes of interest were calculated using the 2^–ΔΔCt^ method, with β-actin used as an internal control. Primer sequences are listed in Supplementary Table 1.

### 2.3 Shotgun metagenomic sequencing

Fecal DNA was extracted from 200 mg aliquots using the PowerSoil DNA Isolation Kit (MoBio Laboratories, United States) following manufacturer’s protocols. Libraries were constructed using the Illumina TruSeq DNA Prep Kit and sequenced on the NovaSeq™ X Plus platform at Novogene Technology Co., Ltd., used nuclease-free water as the negative control.

After quality control of the raw sequencing data (including adapter trimming, quality filtering and de-replication), operational taxonomic units (OTU) clustering was performed with a sequence similarity threshold of 97%. Taxonomic annotation was performed followed by α/β-diversity analyses. Compositional differences were evaluated through: (1) Principal coordinates analysis (PCoA) for community structure visualization; (2) Wilcoxon rank sum test combined with linear discriminant analysis (LDA) effect size (LEfSe) for identifying differentially enriched taxa in different groups; differences were considered statistically significant when *p* ≤ 0.05 and |LDA| value ≥ 2. (3) Microbial co-occurrence networks for elucidating microbial interactions.

### 2.4 Untargeted metabolomics analysis

For each 100 mg of liquid nitrogen-homogenized fecal sample, metabolites were extracted with 500 μL of 80% methanol/water. The mixture was vortexed and incubated on ice for 5 min, then centrifuged at 15,000 *g*, 4 °C for 20 min. The supernatant was diluted to 53% methanol and centrifuged again under the same conditions. The LC-MS/MS analysis of metabolites was performed by UHPLC-Q Exactive HF-X system (Thermo, United States) at Novogene Technology Co., Ltd. The raw data were processed using CD 3.3 for feature extraction and quantification, and then matched with the mzCloud/mzVault/Masslist databases after background subtraction. Metabolites were functionally annotated through KEGG/HMDB/LIPID MAPS. Principal Component Analysis (PCA) and Orthogonal Partial Least Squares Discriminant Analysis (OPLS-DA) multivariate analyses were performed using MetaboAnalyst 6.0^1^ to compare the characteristics of the metabolomics changes between different groups. Multivariate (OPLS-DA with VIP ≥ 1) and univariate analyses (*t*-test, FC ≥ 1.5 or ≤ 0.58 at *p* ≤ 0.05) were used to identify differently expressed metabolites (DEMs), and subsequent pathway analysis and visualization were performed with MetaboAnalyst 6.0 and R.

### 2.5 Statistical analysis

Data are presented as mean ± standard error of the mean (SEM). Statistical analyses were performed using GraphPad Prism (Version 10.1.2) software. Two-way analysis of variance (ANOVA) or unpaired two-tailed Student’s *t*-tests were employed as appropriate. Statistical significance was defined as *p* ≤ 0.05.

## 3 Results

### 3.1 Nicotine alleviated HFD-induced obesity and related metabolic disorders

**FIGURE 1:**
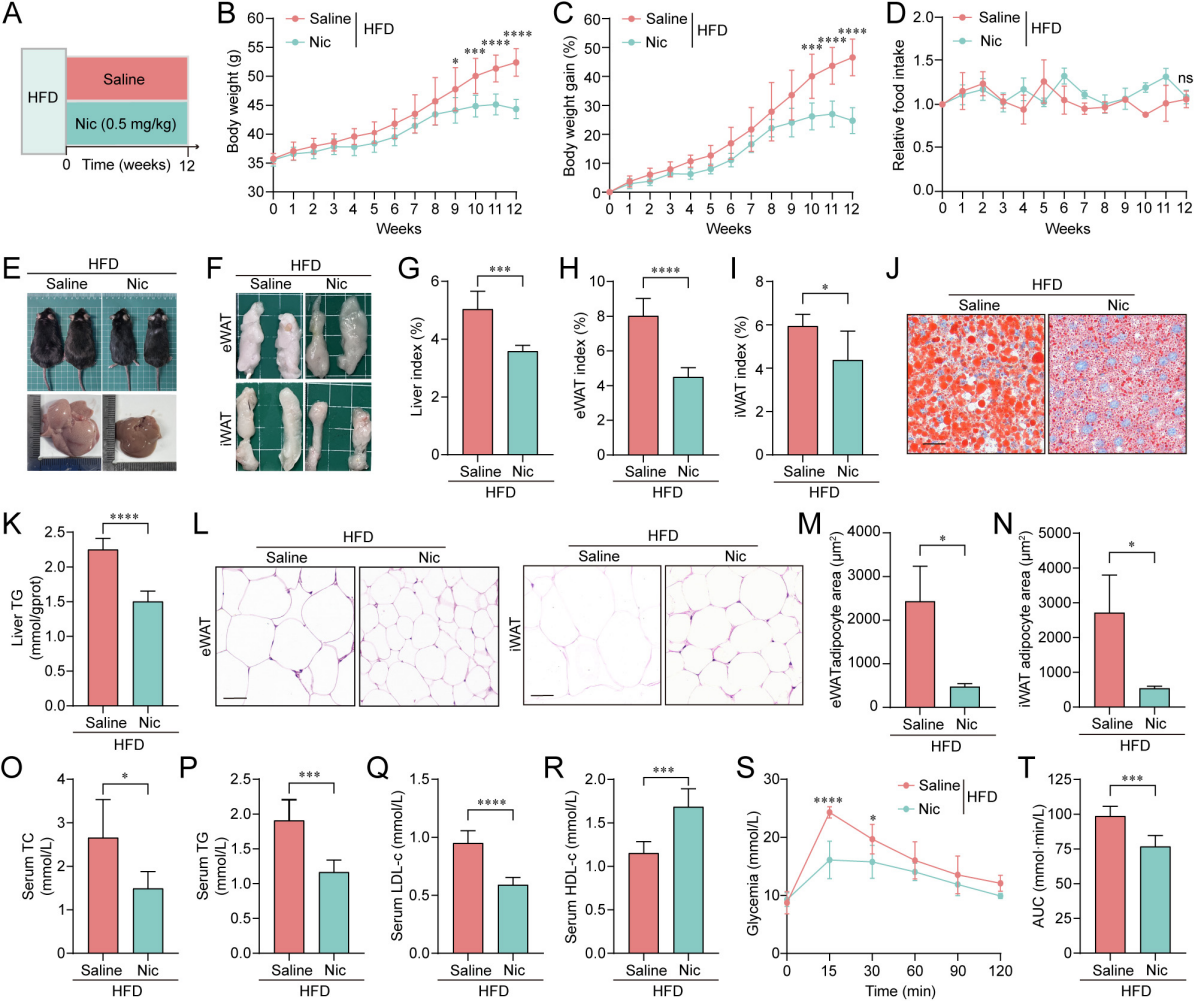
Oral administration of nicotine alleviated high-fat diet (HFD)-induced obesity and related metabolic disorders. **(A)** Experimental timeline: HFD-fed mice were orally administered either saline or nicotine (Nic, 0.5 mg/kg) daily for 12 weeks, *n* = 6 per group. **(B–D)** Body weight **(B)**, body weight gain **(C)**, and relative food intake **(D)** of mice during the 12-week treatment period. **(E)** Representative images of the body size and liver tissue of mice at treatment endpoint. **(F)** Gross morphology of epididymal white adipose tissue (eWAT) and inguinal WAT (iWAT) fat pads. **(G–I)** Quantification of liver index **(G)**, eWAT index **(H)**, and iWAT index **(I)**. **(J)** Oil Red O (ORO) staining of liver sections indicating lipid accumulation (scale bar = 100 μm). **(K)** Liver triglycerides (TG) content. **(L)** Hematoxylin and eosin (H&E) staining of eWAT and iWAT sections (scale bar = 100 μm). **(M,N)** Adipocyte area of eWAT **(M)** and iWAT **(N)** was quantified from three randomized fields for each mouse using Image J. **(O–R)** Serum lipid profiles, including total cholesterol (TC) **(O)**, TG **(P)**, low-density lipoprotein cholesterol (LDL-c) **(Q)**, and high-density lipoprotein cholesterol (HDL-c) **(R)**. **(S,T)** Changes in blood glucose levels **(S)** and corresponding area under the curve (AUC) statistics **(T)** in the oral glucose tolerance test (OGTT) test. All data were presented as mean ± SEM. Line graphs were analyzed by two-way analysis of variance (ANOVA), and bar graphs were analyzed using unpaired two-tailed Student’s *t*-tests. ns, not significant, *p < 0.05, **p < 0.01, ***p < 0.001, ****p < 0.0001.

To investigate the effect of nicotine on HFD-induced obesity, HFD-fed C57BL/6J mice were randomly divided into two groups that were orally administrated nicotine (Nic, 0.5 mg/kg) or saline (negative control) daily for 12 weeks, respectively (Figure 1A). During the course of dosing, the control group showed a significant increase in body weight, while nicotine effectively attenuated HFD-driven weight gains, with minor weight gain from week-8 onward and even weight loss at the end (Figures 1B, C). Notably, the effect of nicotine on suppressing weight gain was independent of food intake (Figure 1D). Furthermore, control mice exhibited typical obesity characteristics with large body size, increased liver volume with steatosis, and fat deposits, including eWAT and iWAT, (Figures 1E, F). Nicotine administration significantly alleviated these obese phenotypes (Figures 1E, F) and reduced the liver and fat indices (Figures 1G–I). The improvement of nicotine on hepatic steatosis was further confirmed by ORO staining of liver sections (Figure 1J) and detection of liver TG content (Figure 1K). Additionally, nicotine administration could significantly mitigate HFD-induced adipocyte enlargement in WAT, as shown by H&E staining analysis (Figures 1L–N).

Given that obesity is generally accompanied by metabolic disorders, we further assessed whether nicotine plays a role in regulating metabolic homeostasis. It was found that HFD-induced dyslipidemia was relieved by nicotine, as evidenced by decreased levels of TC, TG, LDL-c and increased levels of HDL-c in serum (Figures 1O–R). Moreover, nicotine-treated mice had significantly lower glycemia in OGTT test (Figures 1S, T). We also tested diabetes associated indicators and observed decreased glucagon and PAI-1 and increased insulin levels (Supplementary Figures 2A–C), suggesting nicotine restored glucose disposal ability. Further analysis of adipose tissue showed that adipogenesis-related genes (*Acaca*, *Fsn*, *Fabp4*, and *Srebf1*) were downregulated, while lipolysis-related genes (*Lipe* and *Adipoq*) and cholesterol homeostasis regulatory genes (*Ldlr* and *Abca1*) were upregulated by nicotine treatment (Supplementary Figures 2D). Nicotine may also influence lipid absorption through direct or indirect mechanisms, which could contribute to its overall effects on lipid metabolism observed in our study (Aslam et al., 2025). Interestingly, we also found elevated SCFAs that have been reported to have positive effects on amelioration of obesity (Kim, 2023; Supplementary Figures 2E–K), implicating nicotine significantly improved the lipid metabolism. Obesity-induced inflammation tends to exacerbate metabolic disorders, and we also found improvements in inflammatory markers, manifested as the decrease of inflammatory factors TNF-α, IL-1α, IL-6, and chemokine Eotaxin elicited by nicotine (Supplementary Figures 2L–O).

In conclusion, these data demonstrated that oral administration of nicotine exerts protective effects against HFD-induced obesity and associated metabolic dysfunction.

### 3.2 Anti-obesogenic effects of nicotine were dependent on gut microbiota

**FIGURE 2:**
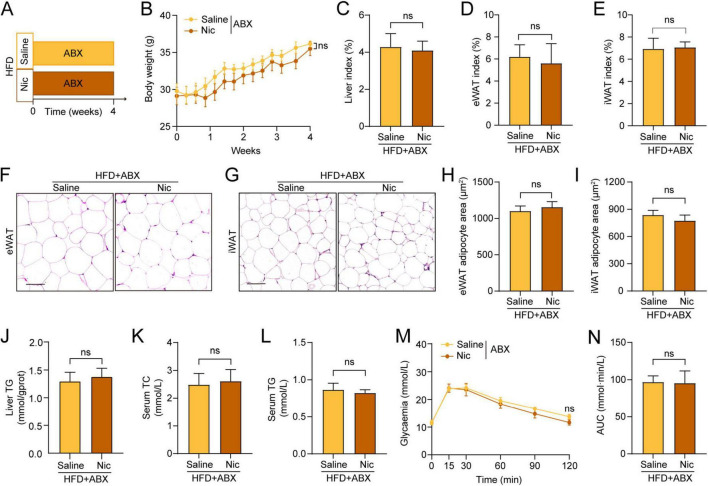
The anti-obesity effects of nicotine were eliminated by antibiotics treatment. **(A)** Experimental illustration: High-fat diet (HFD)-fed mice were treated with a broad-spectrum cocktail of antibiotics (ABX) simultaneously administered either saline or nicotine (Nic, 0.5 mg/kg) daily for 4 weeks, *n* = 6 per group. **(B)** Body weight changes during the treatment period. **(C–E)** Liver index **(C)**, eWAT index **(D)**, and iWAT index **(E)** at treatment endpoint. **(F–I)** Representative images of H&E stained eWAT **(F)** and iWAT **(G)** sections (scale bar = 100 μm), and quantification of adipocyte area in eWAT **(H)** and iWAT **(I)**. **(J)** Hepatic TG content. **(K,L)** Serum levels of TC **(K)** and TG **(L)**. **(M,N)** Glycemia levels at tested points **(M)** and AUC quantification **(N)** in the oral glucose tolerance test (OGTT) test. All data were shown as mean ± SEM. Line graphs were analyzed by two-way analysis of variance (ANOVA), and bar graphs were analyzed using unpaired two-tailed *t*-tests. ns, not significant, *p < 0.05, **p < 0.01, ***p < 0.001, ****p < 0.0001.

Nicotine has been reported to modulate the composition and metabolites of gut microbiota to reduce feeding and body weight (Ohue-Kitano et al., 2024). To determine whether the gut flora mediates the nicotine-induced anti-obesogenic effects under the mode and dose of nicotine administration in our study, we employed ABX and FMT models. HFD mice were exposed to ABX cocktails concurrently with saline or nicotine administration for four consecutive weeks (Figure 2A). It was observed that the ameliorative effects of nicotine on obesity was abolished in the ABX model (Supplementary Figure 3A), with no differences in body weight gain (Figure 2B and Supplementary Figure 3B) between control and nicotine groups, as well as food and water intake (Supplementary Figures 3C, D). Besides, nicotine failed to improve other indications of obesity, including liver index (Figure 2C) and liver steatosis (Supplementary Figures 3E), fat accumulation (Figures 2D, E and Supplementary Figures 3F, G) and adipocyte enlargement (Figures 2F–I). Furthermore, the effects of nicotine on HFD-induced metabolic disorders were also abrogated by antibiotic treatment, as shown by similar liver and peripheral TG and TC levels (Figures 2J–L), together with no difference in glycemic regulatory capacity between two groups (Figures 2M, N).

**FIGURE 3:**
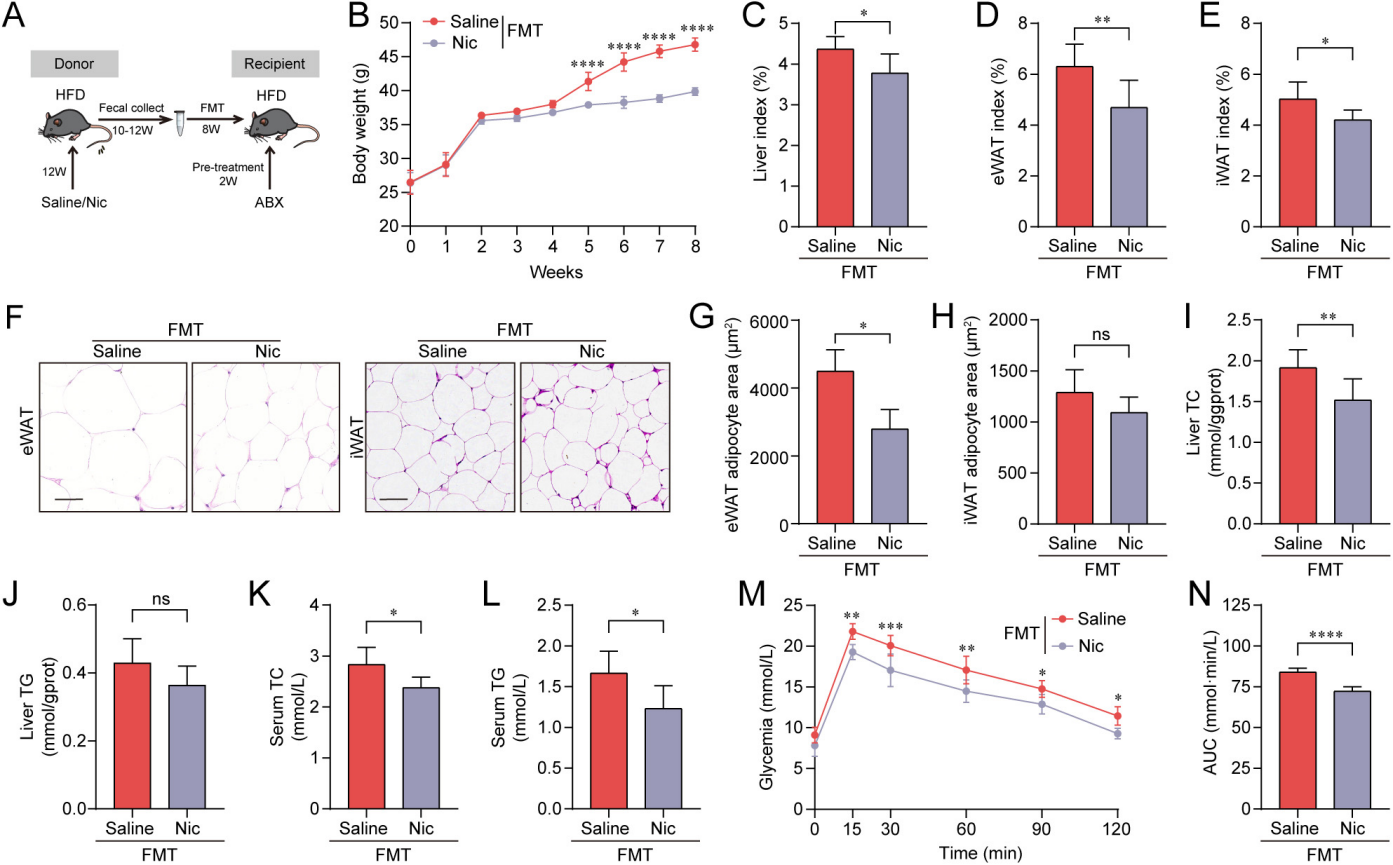
Fecal microbiota transplantation (FMT) from nicotine-treated mice diminished obesity and related metabolic disorders in obese mice. **(A)** Experimental illustration: fecal microbiota from HFD mice given 12-week saline or nicotine were transplanted into ABX-pretreated HFD recipients for 8-week treatment, *n* = 6 per group. **(B)** Bodyweight gain during the treatment period. **(C–E)** Liver index **(C)**, eWAT index **(D)**, and iWAT index. **(F–H)** Representative H&E-stained sections of eWAT and iWAT **(F)** (scale bar = 100 μm), and quantification of adipocyte size in eWAT **(G)** and iWAT **(H)**. **(I,J)** Hepatic TC **(I)** and TG **(J)** content. **(K,L)** Serum levels of TC **(K)** and TG **(L)**. **(M,N)** Variations of blood glucose concentrations post gavage of glucose solution **(M)** and corresponding AUC quantification **(N)** in the oral glucose tolerance test (OGTT) test. All data were presented as mean ± SEM. Line graphs were analyzed by two-way analysis of variance (ANOVA), and bar graphs were analyzed using unpaired two-tailed *t*-tests. ns, not significant, *p < 0.05, **p < 0.01, ***p < 0.001, ****p < 0.0001.

To further verify the role of gut microbiota in nicotine-induced effects, FMT was performed by transplanting fecal microbiota from HFD mice treated with nicotine or saline into HFD recipient mice pre-treated with antibiotics (Figure 3A). It was showed that FMT from nicotine-treated mice could recapitulate the nicotine-induced anti-obesogenic effects. Compared to the control mice, Nic-FMT recipients displayed retarded body weight gain from 2 weeks post transplantation (Figure 3B and Supplementary Figure 4A), lowered liver index (Figure 3C), reduced WAT deposits (Figures 3D, E and Supplementary Figure 4C) and adipocyte area (Figures 3F–H) at the end of treatment, with indistinguishable food intake (Supplementary Figure 4B). In addition, Nic-FMT led to decreased levels of TC and TG in the liver and peripheral circulation (Figures 3I–L), as well as LDL-c levels in serum (Supplementary Figure 4D), although HDL-c levels did not change significantly (Supplementary Figure 4E). Also, glycemic rise was markedly reduced in Nic-FMT mice during the OGTT test (Figures 3M, N), further illustrating that transplantation of nicotine-modified fetal microbiota improved obesity-related metabolic abnormalities. Together, these results confirmed the key role of gut microbiota in the anti-obesogenic effects of nicotine.

### 3.3 Nicotine treatment shifted the gut microbiota of HFD-induced obese mice

**FIGURE 4:**
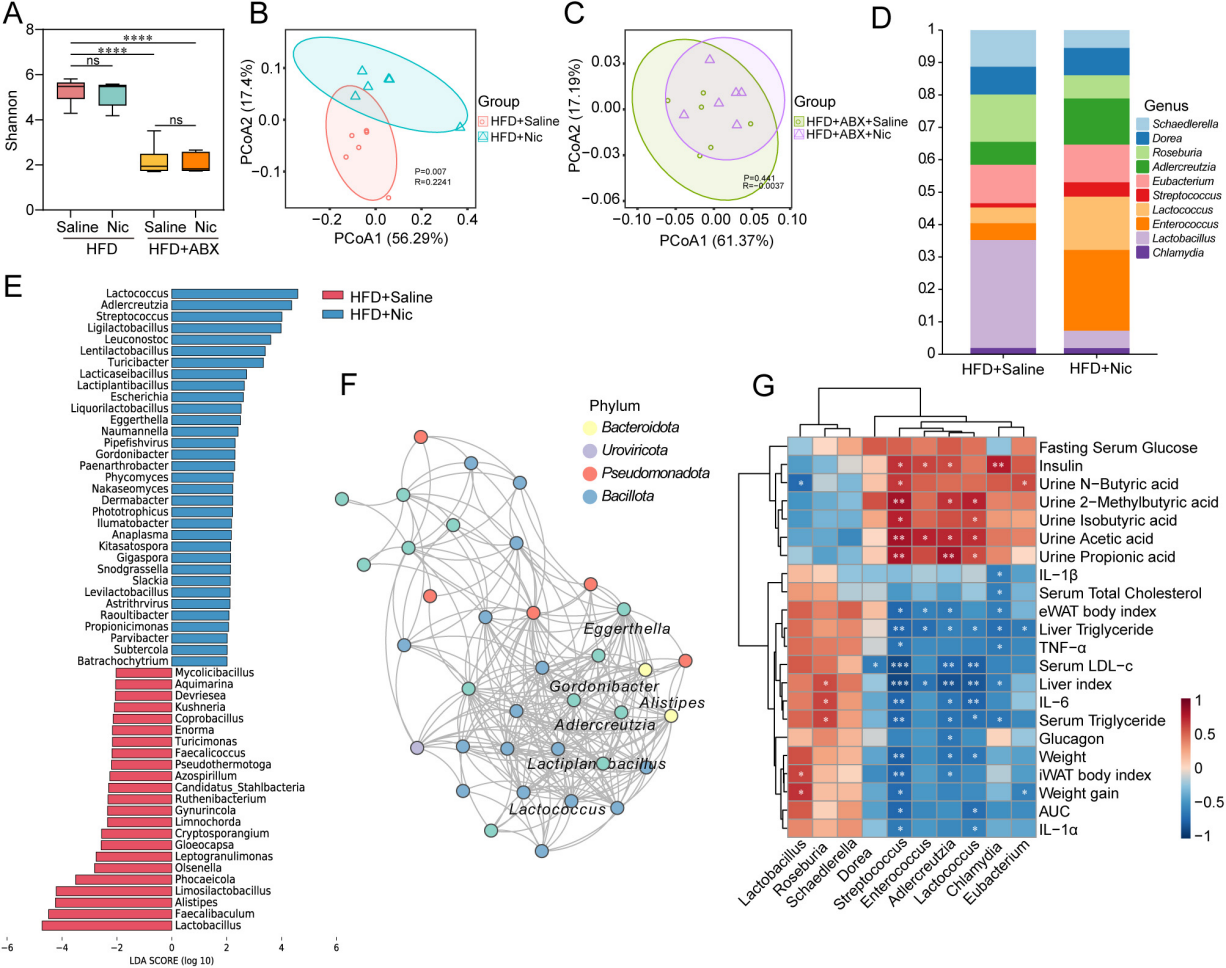
Nicotine administration shifted gut microbiota composition in high-fat diet (HFD) mice. **(A)** α-diversity assessed by Shannon index in four groups. **(B,C)** Principal coordinates analysis (PCoA) plots of β-diversity (weighted UniFrac) for HFD groups **(B)** and HFD + ABX groups **(C)** with or without nicotine treatment. **(D)** Relative abundance of the top 10 bacterial genera at the genus level in HFD mice with or without nicotine treatment. **(E)** LEfSe analysis revealed the bacterial taxa that were differentially abundant (|LDA| value ≥ 2 and *p* ≤ 0.05) in HFD mice with or without nicotine treatment. **(F)** SparCC correlation network of differential taxa shown in panel **(E)** (R > 0.7, *p* < 0.05). **(G)** Spearman’s correlation analysis of specific taxa with obesity-related biochemical indicators at the treatment endpoint. *p < 0.05, **p < 0.01, ***p < 0.001.

To further ascertain the role of gut microbiota in the anti-obesogenic effects of nicotine, changes in the composition of the gut microbiota under nicotine treatment were characterized through metagenomics sequencing. Analysis of α-diversity demonstrated that there were no differences between HFD + Saline and HFD + Nic groups, nor between HFD + ABX + Saline and HFD + ABX + Nic groups. However, compared with HFD groups, the α-diversity of the HFD + ABX groups was significantly reduced, confirming the clearance of gut microbiota under ABX treatment (Figure 4A). Intergroup β-diversity, as visualized by PCoA, showed two separate clusters dependent on nicotine treatment in HFD groups, but not in HFD + ABX groups, indicating differential gut microbial composition in response to nicotine interventions (Figures 4B, C). For the top 10 abundant microbiota at the genus level, nicotine treatment significantly increased levels of *Adlercreutzia*, *Chlamydia*, *Dorea*, *Eubacterium*, *Lactococcus* and *Streptococcus*, while decreased levels of *Enterococcus*, *Lactobacillus*, *Roseburia* and *Schaedlerella* (Figure 4D).

Linear discriminant analysis (LDA) effect size analysis was performed to identify bacterial taxa that shifted by nicotine. At the genus level, compared to the HFD control group, there were 56 genera shown to be changed by nicotine (33 genera increased and 23 genera decreased) (Figure 4E). We constructed a SparCC microbial network of these bacterial genera, and identified six hub taxa (Figure 4F). By comparing the differential microbiota between HFD + Nic and HFD groups, and that between HFD + Nic and HFD + ABX + Nic groups, seven common differential genera were found (Supplementary Figures 5A, B). It was noted that *Adlercreutzia* and *Lactococcus* were not only common differential taxa but also central taxa, and were significantly upregulated by nicotine. Additionally, spearman’s correlation analysis indicated that *Adlercreutzia* and *Lactococcus* were significantly and negatively correlated to multiple biochemical indicators related to obesity, and positively correlated to several SCFAs levels (Figure 4G). These results suggested that nicotine modulates HFD-induced obesity in a gut microbiota-specific manner, and *Adlercreutzia* and *Lactococcus* may be key factors. Given that *Lactococcus* is a well-recognized probiotic and widely used in food, we mainly focus on the function of *Adlercreutzia*.

### 3.4 Adlercreutzia was a key factor for nicotine reducing HFD-induced obesity

**FIGURE 5:**
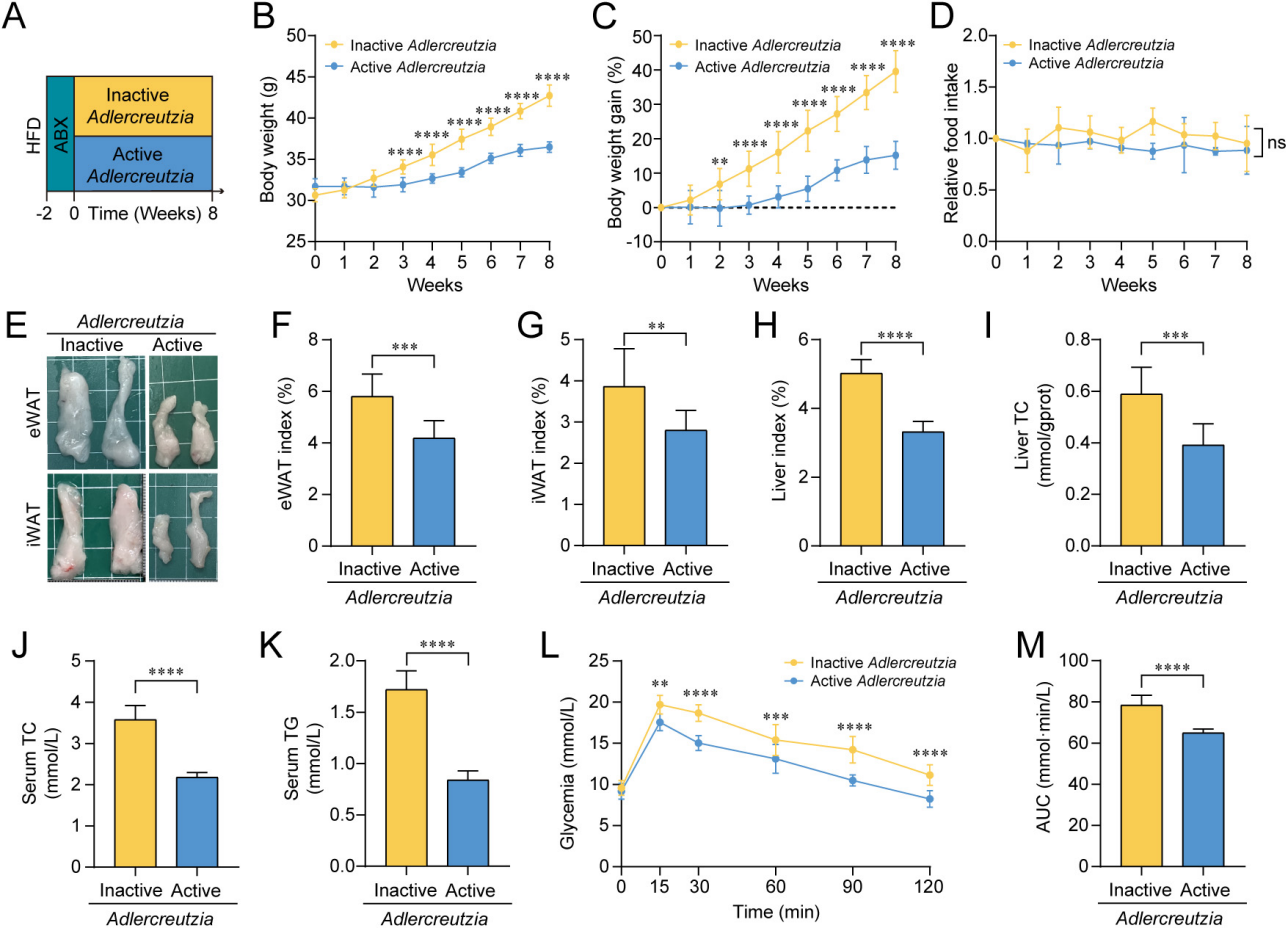
Active *Adlercreutzia* transplantation ameliorated high-fat diet (HFD)-induced obesity and related metabolic disorders. **(A)** Experimental timeline: HFD-fed mice were pretreated with ABX for 2 weeks and subsequently transplanted with either heat-inactivated or active *Adlercreutzia* for 8 weeks, *n* = 6 per group. **(B–D)** Changes of body weight **(B)**, percent body weight gain **(C)** and relative food intake during the treatment period **(D)**. **(E–G)** Gross morphology of eWAT and iWAT fat pads **(E)**, as well as eWAT index **(F)** and iWAT index **(G)**, respectively. **(H,I)** Liver index **(H)** and hepatic TC levels **(I)**. **(J,K)** Serum TC **(J)** and TG **(K)** concentrations. **(L,M)** Curve of blood glucose levels over time **(L)** and quantification of area under the curve (AUC) **(M)** in the oral glucose tolerance test (OGTT) test. Data were presented as mean ± SEM. Line graphs were analyzed by two-way analysis of variance (ANOVA), and bar graphs were analyzed by unpaired two-tailed Student’s *t*-tests. ns, not significant, *p < 0.05, **p < 0.01, ***p < 0.001, ****p < 0.0001.

To investigate whether *Adlercreutzia* can produce anti-obesogenic effects in HFD mice, *Adlercreutzia equolifaciens* was chosen as a representative of the genus due to its well-characterized metabolic capabilities and experimental tractability, with a metabolic profile consistent with the lipid-related patterns observed in our study (Maruo et al., 2008; Tian and Bisanz, 2022). HFD recipients pre-treated with antibiotics were orally gavaged with either active or heat-inactivated *Adlercreutzia* daily for 8 weeks (Figure 5A). Mice colonized with active *Adlercreutzia* exhibited significantly slower body weight gain (Figures 5B, C) compared to the inactive group, despite similar food intake across groups (Figure 5D). Treatment with active *Adlercreutzia* also effectively alleviated other HFD-induced obesity traits, as shown by reduced fat deposition (Figures 5E–G), lowered liver index accompanied by decreased hepatic TC content (Figures 5H, I), as well as reduced serum TC and TG levels (Figures 5J, K). Regarding the OGTT test, mice transplanted with active *Adlercreutzia* showed greater glucose tolerance compared to the control mice, with lower blood glucose concentrations at multiple time points post glucose gavage, as well as less blood glucose accumulation as shown by AUC (Figures 5L, M). Overall, these results demonstrated that colonization with active *Adlercreutzia* protects mice against HFD-induced obesity, dyslipidemia, and impaired glycemic regulation, further confirming that *Adlercreutzia* is a key mediator for the anti-obesity effects of nicotine.

### 3.5 Nicotine altered lipid metabolism via gut microbiota-metabolite axis in obese mice

**FIGURE 6:**
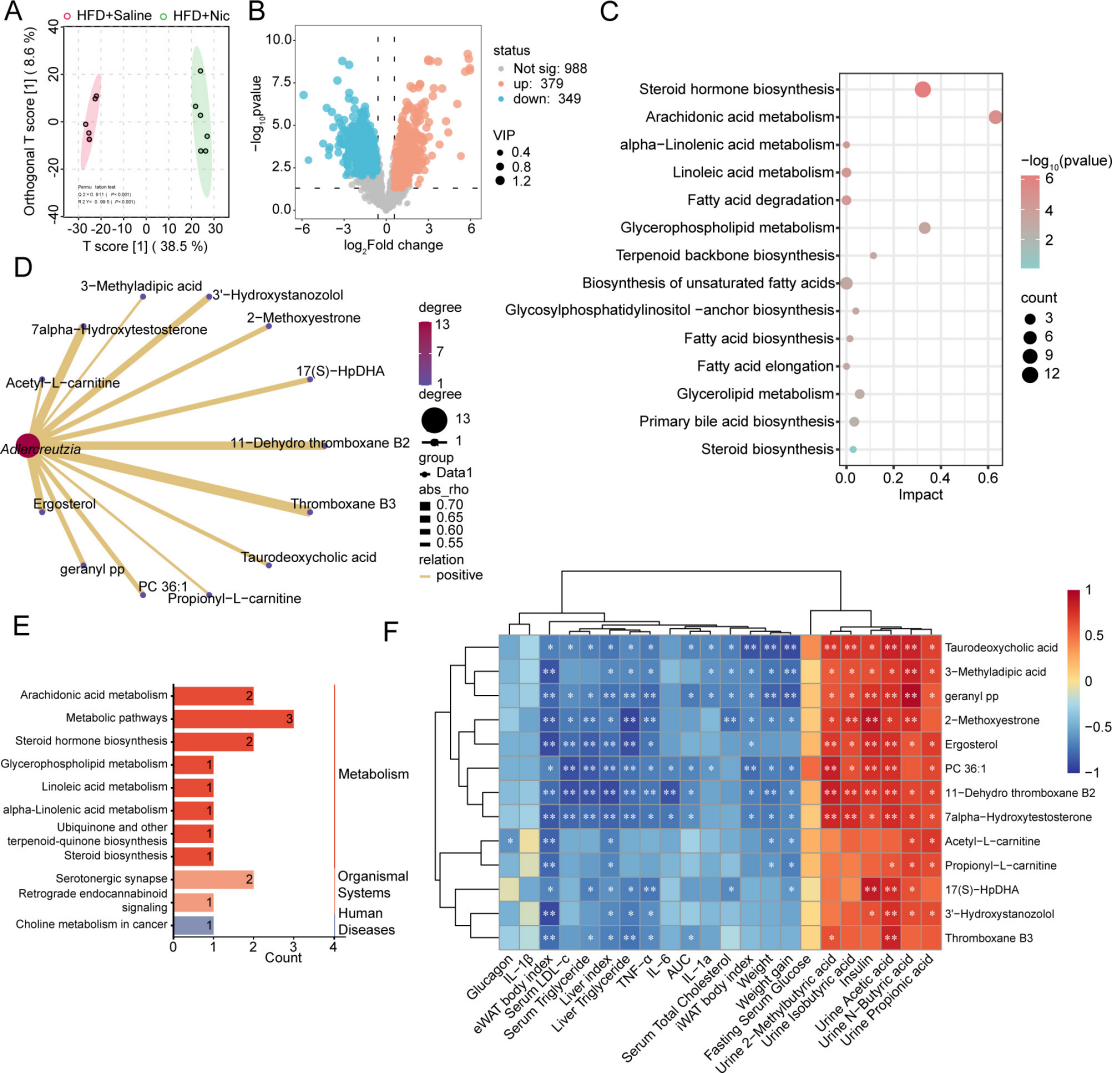
Nicotine intervention altered metabolic profiles via gut microbiota-metabolite axis in obese mice. **(A)** Orthogonal partial least squares discriminant analysis (OPLS-DA) revealed a clear separation between HFD + Saline and HFD + Nic groups, indicating distinct metabolic profiles upon nicotine treatment. **(B)** Volcano plot showed differently expressed metabolites (DEMs) including 379 upregulated (orange) and 349 downregulated (blue) metabolites (VIP ≥ 1.0, FC ≥ 1.5 or ≤ 0.58 and *p* ≤ 0.05) in HFD + Nic group compared to HFD + Saline group. **(C)** Metabolic pathway enrichment analysis on DEMs related to lipids and lipid-like molecules using MetaboAnalyst 6.0 online tool. **(D)** Lipid metabolites screened by spearman’s correlation analysis of *Adlercreutzias* with lipid-related differently expressed metabolites (DEMs), with correlation coefficients (R) > 0.5, FC > 2, and positive correlation with *Adlercreutzia*. **(E)** KEGG pathway enrichment analysis on lipid metabolites shown in panel **(D)**. **(F)** Spearman’s correlation analysis of lipid metabolites regulated by *Adlercreutzias* with biochemical indicators related to obesity. *p < 0.05, **p < 0.01, ***p < 0.001, ****p < 0.0001.

We further explored whether gut microbiota-derived metabolites were implicated in the nicotine-induced anti-obesity effects through untargeted metabolomics analysis. PCA analysis showed that the QC samples gathered together, indicating stability of sequencing and data availability (Supplementary Figures 6A). OPLS-DA showed separate clusters of HFD and HFD + Nic groups (Figure 6A), suggesting differences in metabolic profiles in response to nicotine treatment. A total of 1,716 metabolites were identified in two groups of 12 samples, and metabolite classification enrichment analysis revealed that these metabolites mainly were lipids and lipid-like molecules (Supplementary Figures 6B). Differently expressed metabolites (DEMs) between two groups were screened (VIP ≥ 1.0, FC ≥ 1.5 or ≤ 0.58 and *p* ≤ 0.05), and identified 728 DEMs, of which, 379 were upregulated and 349 were downregulated (Figure 6B). KEGG pathway enrichment analysis of 337 HMDB-matched DEMs among all DEMs showed that these DEMs were involved in extensive metabolic pathways related to amino acid, vitamin, lipid and carbohydrate metabolism (Supplementary Figures 6C). Since we are more concerned with the DEMs related to lipids and lipid-like molecules, further KEGG pathway enrichment analysis on 182 such DEMs was conducted, and found they were predominantly involved in pathways including Steroid hormone biosynthesis, Arachidonic acid metabolism, α-Linolenic acid and Linoleic acid metabolism, Fatty acid degradation and Glycerophospholipid metabolism (Figure 6C).

To find out lipid metabolites regulated by *Adlercreutzia*, spearman’s correlation analysis of *Adlercreutzias* with lipid-related DEMs was performed, and lipid metabolites with correlation coefficients (R) > 0.5, FC > 2, and positively correlated with *Adlercreutzia* were screened (Figure 6D). Among 13 identified lipid metabolites, 11-Dehydro thromboxane B2, 2-Methoxyestrone, 7α-Hydroxytestosterone, Ergosterol, geranyl pp, and PC 36:1 were involved in pathways such as Arachidonic acid metabolism, Serotonergic synapse, Steroid hormone biosynthesis, Glycerophospholipid metabolism, Linoleic acid and α-Linolenic acid metabolism (Figure 6E). In addition, the correlation analysis of the six lipid metabolites with biochemical indicators related to obesity indicated that these metabolites associated with improved obesity characteristics (Figure 6F).

Further screening of lipid-related DEMs that co-regulated by *Adlercreutzia* and *Lactococcus* identified seven major metabolites: Acetyl-L-carnitine, Ergosterol, geranyl pp, PC 36:1, Propionyl-L-carnitine, 3’-Hydroxystanozolol, and 17(S)-HpDHA (Supplementary Figure 6D). These metabolites were similarly engaged in steroid synthesis and PUFA metabolism (Supplementary Figure 6E). Collectively, these findings suggested nicotine modulates lipid metabolism in obesity via gut microbiota-metabolite axis. The metabolism of PUFA regulated by *Adlercreutzia* and *Lactococcus* may be closely related to the anti-obesity effects of nicotine.

## 4 Discussion

This study systematically evaluated the effects of oral low-dose nicotine administration on high-fat diet (HFD)-induced obesity and metabolic abnormalities, revealing a critical regulatory role of the gut microbiota. To directly target the gut microbiota and simultaneously preclude addictive effects, we administered nicotine orally at a dose of 0.5 mg/kg. This dosage is both below the established addiction threshold (0.5–2.0 mg/kg) for intraperitoneal injection in mice (Matta et al., 2007; Ohue-Kitano et al., 2024) and lower than the range typically used in metabolic studies (1.5–4.0 mg/kg), thus allowing the isolation of its pure regulatory effects. The oral route was chosen based on evidence that it directly influences microbial communities via mechanisms including increased intestinal pH (Tomoda et al., 2011).

The results showed that continuous oral administration of nicotine at a low, sub-addictive dose (0.5 mg/kg) for 12 weeks significantly suppressed body weight gain in HFD-fed mice. Notably, there was no significant difference in food consumption, suggesting that the anti-obesity effect of nicotine was independent of energy intake and may not involve relevant central mechanisms (Li et al., 2025). The weight reduction was accompanied by a decrease in adipose tissue mass, a reduction in liver index, and marked improvement in hepatic steatosis as well as glucose and lipid metabolism abnormalities. It has reported (Qin et al., 2025) that repeated subcutaneous administration of low and moderate-dose nicotine (0.5 and 1 mg/kg) results in more pronounced body weight reduction compared to a single injection. In contrast, under acute administration, even when the dose exceeds 4 mg/kg, it only reduces liver weight without improving hepatic morphology or lipid accumulation. These findings highlight the importance of nicotine dosage, administration route and duration in determining its metabolic effects. Our study further confirmed that oral administration of chronic low-dose nicotine not only reduced body weight and fat deposit but also had a favorable effect on hepatic lipid metabolism in obese mice.

In this study, metagenomic sequencing revealed that nicotine intervention significantly reshaped the gut microbiota composition in HFD-fed mice, with a notable enrichment of potential probiotic genera, *Adlercreutzia* and *Lactococcus*, and reduced levels of potentially pathogenic genera such as *Enterococcus* and *Roseburia*. *Adlercreutzia* showed strong associations with metabolic indicators. Its abundance was negatively correlated with body weight, adiposity index, liver index and triglycerides (TG) content, and multiple lipid marker levels in plasma, while positively correlated with short-chain fatty acids (SCFAs, such as acetate and propionate) and insulin levels, suggesting a central role in nicotine-mediated metabolic improvement. *Adlercreutzia* belongs to the *Actinobacteriota* phylum and has attracted much attention for its ability to produce SCFAs (e.g., acetate) and metabolize dietary polyphenols. Previous study has shown that *Adlercreutzia equolifaciens* can convert soy isoflavones into equol, a metabolite with estrogen-like activity, which helps to enhance insulin sensitivity, reduce inflammation, and improve lipid metabolism (Vázquez et al., 2021). In HFD-induced obesity models, several studies (Jian et al., 2025; Lu et al., 2021) have reported a positive association between the abundance of *Adlercreutzia* and improvements of obesity indicators. For instance, Ohue-Kitano et al. (2024) demonstrated that supplementation with gallic acid (GA) significantly increased the abundance of *Adlercreutzia*, accompanied by reductions in body weight and improvements in glucose tolerance, insulin resistance, as well as blood glucose and lipid parameters. In this study, we confirmed and functionally validated the pivotal mediating role of *Adlercreutzia* specifically under nicotine intervention, which extend the understanding of the role of *Adlercreutzia* in metabolic health and strengthen the evidence for *Adlercreutzia* as a promising probiotic candidate against obesity and metabolic disorders.

Nicotine significantly influenced multiple metabolic pathways, particularly those involved in steroid biosynthesis, glycerophospholipid metabolism, and unsaturated fatty acids (PUFA) metabolism. Several lipid metabolites were found to be strongly associated with *Adlercreutzia*, including 2-methoxyestrone, ergosterol, 7α-hydroxytestosterone, and PC 36:1, suggesting that this genus may exert regulatory effects by modulating steroid and PUFA metabolism. Notably, PUFA such as arachidonic acid, linoleic acid, and α-linolenic acid are not only precursors of inflammatory mediators but also closely associated with energy metabolism and adipose tissue inflammation. Dysregulation of these fatty acids is commonly observed in obesity with insulin-resistant status (Miyamoto et al., 2019). In addition, ergosterol, although a fungal-derived sterol, has gradually drawn attention for its metabolism and signaling role in the host’s gut. Emerging evidence suggests that ergosterol may regulate lipid metabolism and inflammatory responses through pathways such as PPARγ signaling (Das and Gurusiddaiah, 2023), and its potential involvement in metabolic remodeling warrants further investigation.

In contrast to previous studies emphasizing the central and peripheral mechanisms by which nicotine regulates feeding behavior and body weight (Li et al., 2025), our study reveals that nicotine can act on the gut microbiota and improve obesity through microbiota-derived metabolites. Importantly, we adopted a therapeutic intervention model in which nicotine treatment was initiated after the establishment of obesity, thereby highlighting its remedial, rather than merely preventive effect. Moreover, the low-dose oral gavage regimen effectively minimized the risk of nicotine addiction and related side-effects. Previous study (Ohue-Kitano et al., 2024) has reported that intraperitoneal injection of nicotine at 1.5 mg/kg indirectly modulated the gut microbiota via nicotinic acetylcholine receptors and increased the abundance of the genus *Lactobacillus* and the levels of metabolite KetoB, a fatty acid derivative, which was shown to have anti-obesity potential. In contrast, our study observed a decrease in *Lactobacillus* abundance, while oral nicotine administration selectively enriched *Lactococcus* and *Adlercreutzia*. This discrepancy may be attributed to the difference in administration routes. Intraperitoneal injection allows nicotine to be rapidly absorbed through peritoneal capillaries and distributed systemically, whereas oral gavage delivers nicotine directly through the gastrointestinal tract, making it more suitable for gut microbiota-related studies in chronic conditions such as obesity.

Nevertheless, there are limitations for our study. For instance, multiple dose groups were not set up to systematically analyze the dose-response relationship, and the impacts on the intestinal microbiota. Although the critical role of *Adlercreutzia* was confirmed through transplantation, its precise molecular mechanisms have not been fully revealed. Whether its effects involve host immune recognition, intestinal barrier remodeling, or specific microbial metabolites still requires further investigation. Future validation and mechanistic studies with larger cohorts that include both male and female mice are planned to strengthen and extend our findings, in particular to assess the sex-dependent effects on gut microbiota, metabolism, and treatment responses (Jiang et al., 2025; Kaliannan et al., 2018; Whitehead et al., 2022). Finally, although including an HFD group without antibiotics (ABX) would allow a more comprehensive assessment of ABX’s independent effects, the primary aim of this study was mechanistic validation, and the impact of ABX on basal metabolism has been well-documented in previous studies; therefore, it was not examined here.

In summary, our study demonstrates that oral nicotine administration exerts anti-obesity effects by reshaping the gut microbiota and its associated metabolites, thereby improving host lipid metabolism and alleviating low-grade inflammation. These findings not only expand our understanding of the metabolic actions of nicotine but also highlight *Adlercreutzia* as a promising microbial target for the development of novel microbiota-based interventions for obesity and related metabolic disorders.

## 5 Conclusion

Overall, our study demonstrates that oral administration of nicotine significantly alleviates high-fat diet-induced obesity and metabolic disorders in mice through a gut microbiota–dependent manner. Among the microbial taxa altered by nicotine, the genus *Adlercreutzia* was significantly enriched and strongly associated with improved metabolic outcomes. Functional validation confirmed that *Adlercreutzia* alone is sufficient to reproduce the anti-obesogenic effects of nicotine, highlighting its role as a key mediator of nicotine’s metabolic benefits. In parallel, untargeted metabolomics showed that nicotine profoundly remodeled host lipid metabolism via the gut microbiota–metabolite axis, particularly affecting pathways related to steroid biosynthesis and polyunsaturated fatty acid metabolism. These findings uncover a novel microbiota-mediated mechanism underlying the anti-obesity effects of nicotine and identify *Adlercreutzia* as a promising microbial target for the development of microbiome-based therapies against obesity and related metabolic disorders.

## Data Availability

All sequencing data generated in this study have been submitted to the NCBI under accession number PRJNA1285319.

## Footnotes

1
https://www.metaboanalyst.ca/
