# Supplementary figures and images for: Adlercreutzia-modulated polyunsaturated fatty acid metabolism underlies nicotine’s anti-obesity effects
Source: Front Microbiol. 2025 Dec 18;16:1682370. doi: 10.3389/fmicb.2025.1682370 (PMC12756887; doi:10.3389/fmicb.2025.1682370)

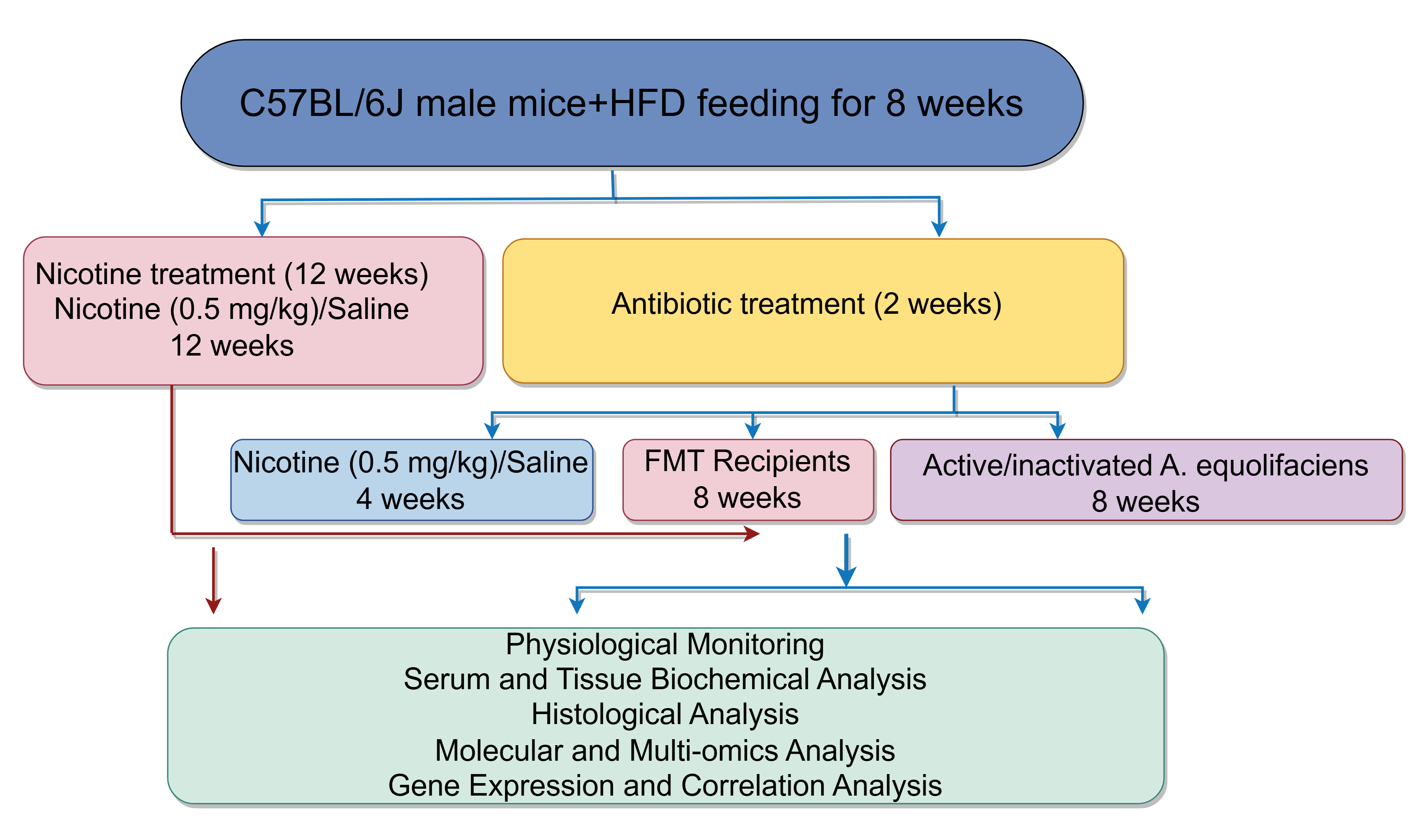

Supplement: Supplementary file 1 [file Image_1.tif]

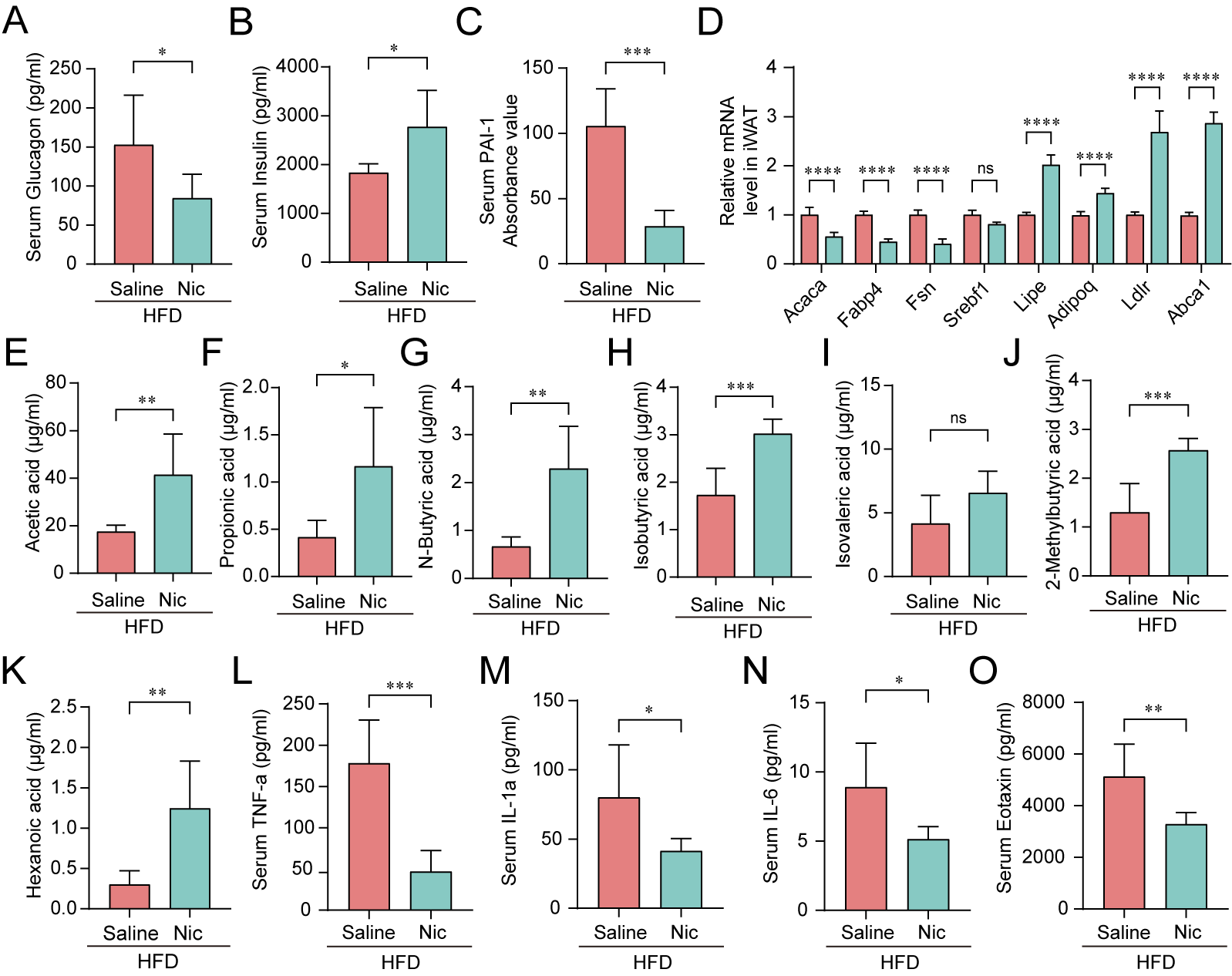

Supplement: Supplementary file 2 [file Image_2.tif]

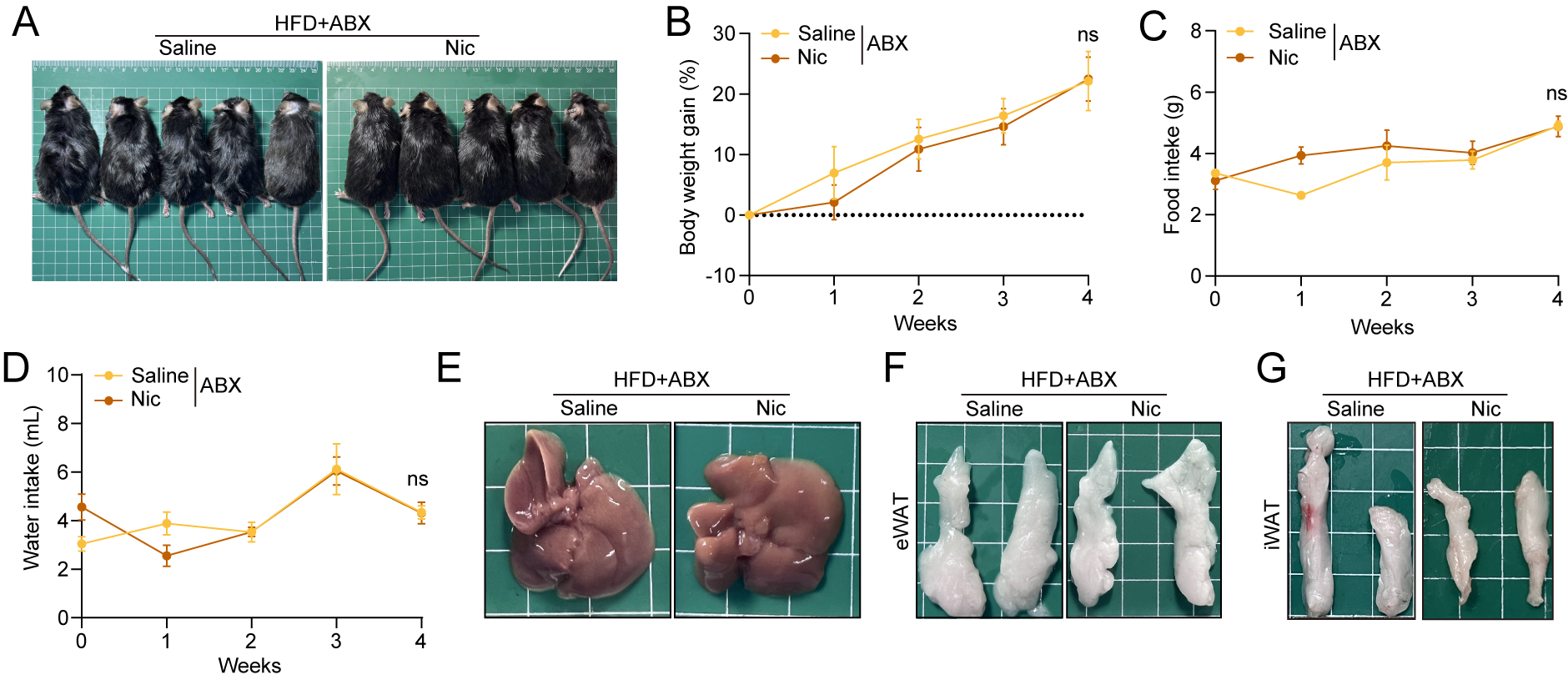

Supplement: Supplementary file 3 [file Image_3.tif]

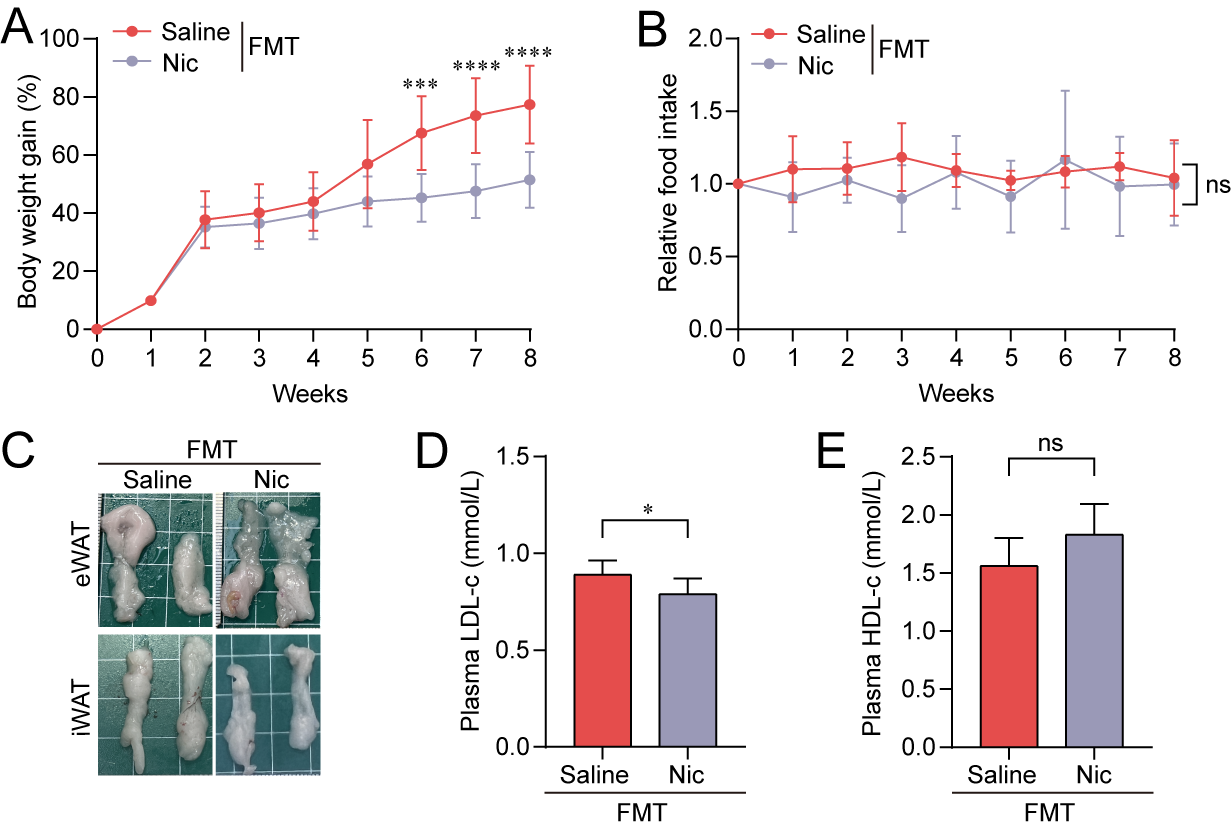

Supplement: Supplementary file 4 [file Image_4.tif]

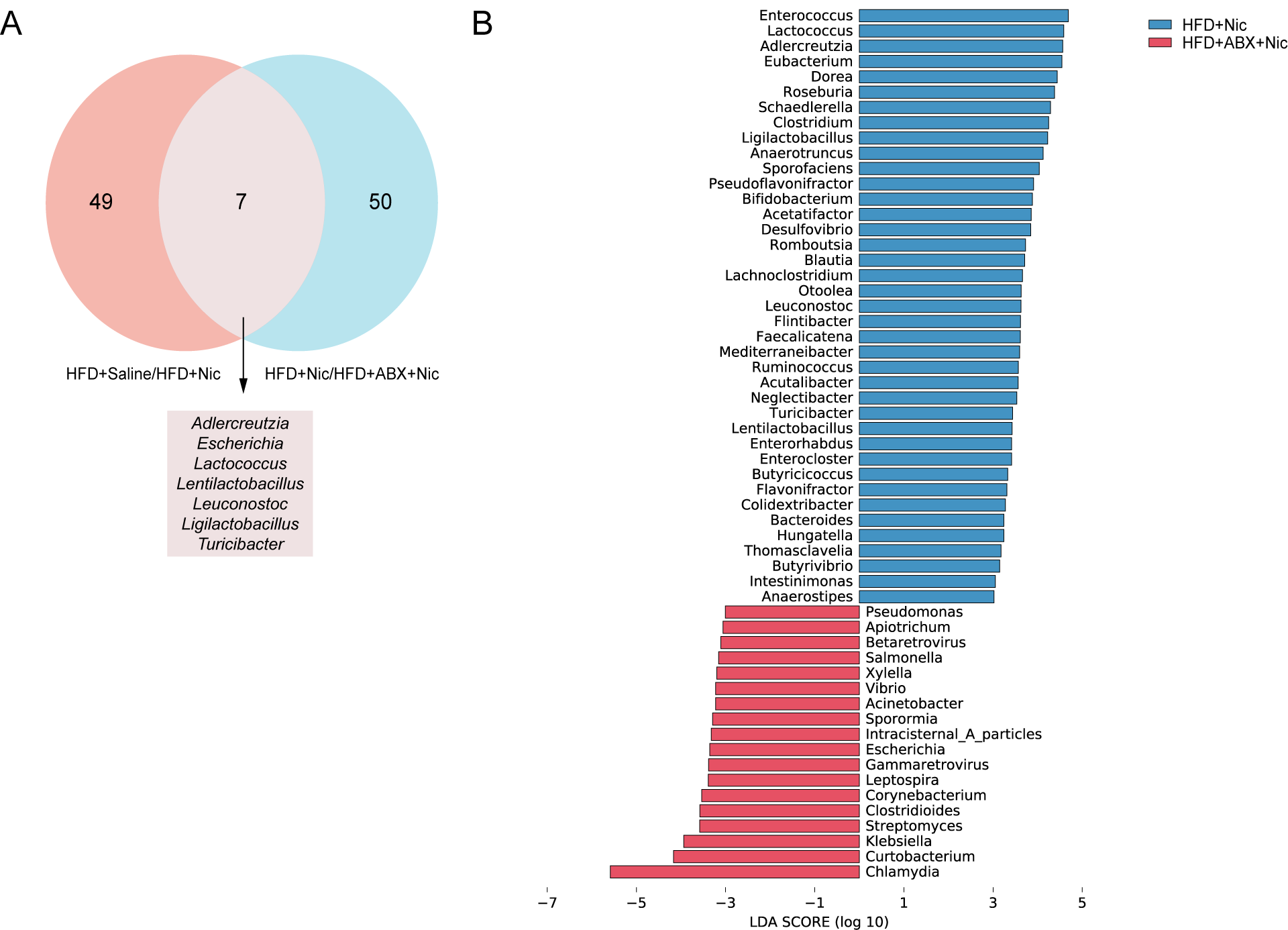

Supplement: Supplementary file 5 [file Image_5.tif]

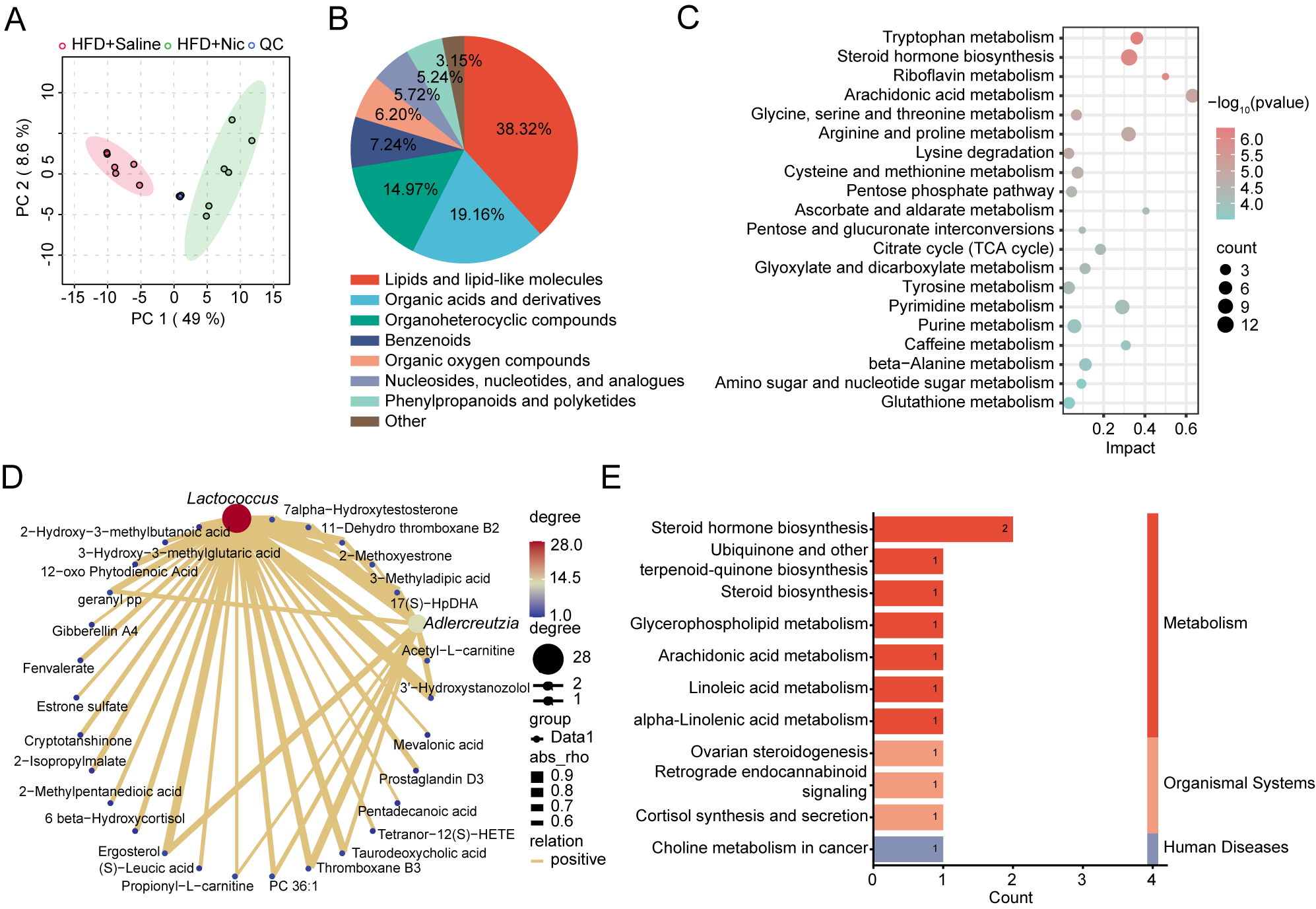

Supplement: Supplementary file 6 [file Image_6.tif]
