# Supplementary material for: Adlercreutzia-modulated polyunsaturated fatty acid metabolism underlies nicotine’s anti-obesity effects
Source: Front Microbiol. 2025 Dec 18;16:1682370. doi: 10.3389/fmicb.2025.1682370 (PMC12756887; doi:10.3389/fmicb.2025.1682370)
Supplement: Supplementary file 7 [file Table_1.docx]

**Table S1**

| Gene | Forward Primer (5′→3′) | Reverse Primer (5′→3′) |
| --- | --- | --- |
| *Acaca* | GCAGCTCTGGAGGTGTATGT | TTCTGTTTAGCGTGGGGATGT |
| *Fabp4* | TCACCATCCGGTCAGAGAGTA | TCCTGTCGTCTGCGGTGATT |
| *Fsn* | GGCTTGTGGGAAGTCAGCCTA | ACTACCGTGGCAAAGTGTTCT |
| *Srebf1* | CCTGCACTTCTTGACACGTT | CAGTCCCCGTCCACAAAGAA |
| *Lipe* | CAGTGCCTATTCAGGGACAGA | TGTGGGCGATGTGGTCTTTT |
| *Adipoq* | AGAGAAAGGAGATGCAGGTCTTC | AAGCGAATGGGTACATTGGGA |
| *Ldlr* | GCCAGTGTGACCGTGAACAT | CACTCGTTGGTCTTGCACTCC |
| *Abca1* | GCTACCCACCCTACGAACAA | ACAGGCGAGACACGATGGAC |
| *Actb* | CATCCGTAAAGACCTCTATGCCAAC | ATGGAGCCACCGATCCACA |
